# Midterm outcomes of simultaneous carotid revascularization combined with coronary artery bypass grafting

**DOI:** 10.1186/s12872-022-02986-y

**Published:** 2022-12-08

**Authors:** Songhao Jia, Maozhou Wang, Ming Gong, Hongjia Zhang, Wenjian Jiang

**Affiliations:** Department of Cardiac Surgery, Beijing Anzhen Hospital, Capital Medical University, Beijing, 100029 China

**Keywords:** Carotid endarterectomy, Carotid artery stenting, Coronary artery bypass grafting, Mid-term outcome

## Abstract

**Background:**

Simultaneous carotid endarterectomy (CEA) combined with coronary artery bypass grafting (CABG) has been widely used in patients with coronary heart disease complicated with severe carotid stenosis to reduce the risk of stroke and death. Carotid artery stenting (CAS) has been proven to be an alternative to CEA in recent years. We investigated the early and mid-term outcomes of simultaneous CEA or CAS combined with CABG in these patients.

**Methods:**

From January 2011 to January 2021, 88 patients who underwent simultaneous carotid revascularization combined with CABG under the same anesthesia in Beijing Anzhen Hospital were retrospectively analyzed, and this study included 25 patients who underwent CAS–CABG and 63 patients who underwent CEA–CABG. The main outcomes included all-cause death, stroke, myocardial infarction and combined adverse events. The main outcomes of the two groups were compared at 30 days after the operation and the mid-term follow-up. Univariate and multivariate Cox proportional hazards regression analyses were performed to determine the independent risk factors affecting mid-term mortality.

**Results:**

Within 30 days after the operation, there was no significant difference in combined adverse events between the two groups (*P* = 0.88). During the median follow-up period of 6.69 years (IQR, 5.82–7.57 years), 9 patients (14.30%) in the combined CEA–CABG group died, while 1 patient (4.00%) in the combined CAS–CABG group died. There were no significant differences in mid-term death (*P* = 0.20), stroke (*P* = 0.78), myocardial infarction (*P* = 0.88), or combined adverse events (*P* = 0.62) between the two groups. Univariate and multivariate Cox proportional hazards regression showed that NYHA grade IV (HR 5.01, 95% CI 1.16–21.64, *P* = 0.03) and previous myocardial infarction (HR 5.43, 95% CI 1.01–29.29, *p* = 0.04) were independent risk factors for mid-term mortality. We also found that combined CEA–CABG surgery may be associated with a higher risk of death (HR, 13.15; 95% CI 1.10–157.69, *p* = 0.04).

**Conclusions:**

Combined CAS–CABG is a safe and effective treatment for patients with coronary heart disease complicated with severe carotid stenosis. NYHA grade IV and previous MI were independent risk factors for mid-term mortality.

## Introduction

Coronary heart disease is one of the most common causes of death in the world [1]. As a result of the progression of systemic atherosclerosis, many patients with coronary heart disease also suffer from carotid artery stenosis. The prevalence of severe carotid artery disease in patients undergoing coronary artery bypass grafting (CABG) is approximately 6% to 14% [2, 3]. Perioperative stroke is one of the most serious complications of CABG, and its mortality can reach 24.8% [4]. Previous studies have confirmed that severe carotid stenosis is an independent risk factor for perioperative stroke in patients undergoing CABG [5]. Previous studies suggested that carotid endarterectomy (CEA) or carotid stent implantation (CAS) should be performed before cardiac surgery (stage) or at the same time (combined) to reduce stroke or death after CABG [6–8]. In the absence of randomized controlled trials, the best treatment for severe carotid stenosis in the CABG population is still controversial. Among American patients who underwent combined carotid revascularization and CABG, combined CEA–CABG was the most frequently performed procedure, followed by staged CEA–CABG [9]. Recently, CAS has been proven to be an alternative to CEA [10]. However, due to the small number of patients receiving combined CAS–CABG, there is still a lack of research comparing the clinical outcomes of combined CEA–CABG and combined CAS–CABG. This paper compares the early and mid-term results of simultaneous carotid revascularization combined with CABG. We conducted this study through a retrospective cohort study with the primary research objective of exploring the efficacy of simultaneous carotid revascularization combined with CABG surgery and the secondary research objective of exploring the risk factors affecting the prognosis of patients undergoing the combined procedure. We present the content of the article according to the STROBE Checklist.

## Methods

### Participants and definitions

**Fig. 1:**
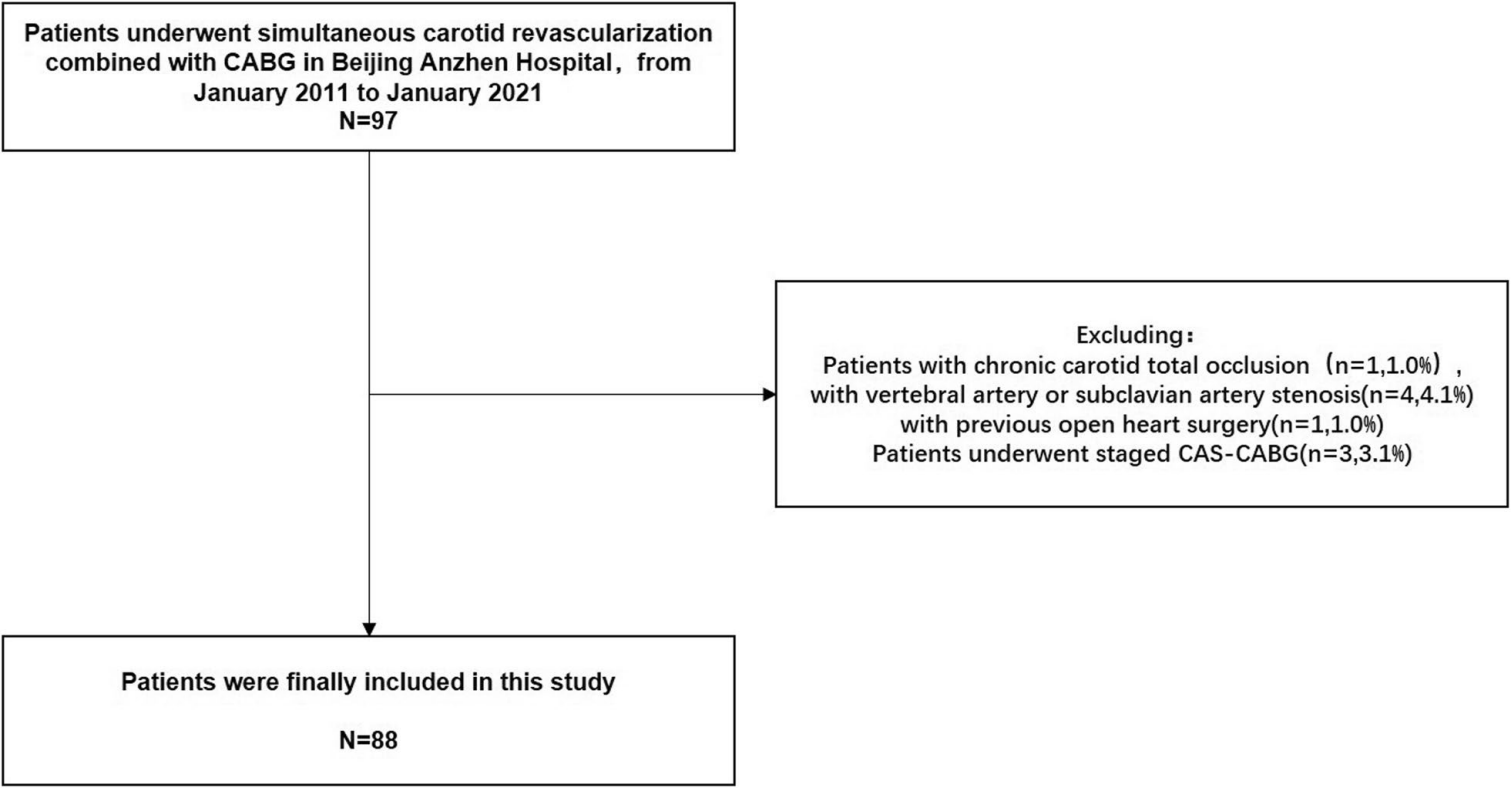
Flow chart of the screening and enrollment of study patients. CABG, Coronary artery bypass graft; CAS, coronary artery stenting

From January 2011 to January 2021, 97 patients underwent simultaneous carotid revascularization combined with CABG at Beijing Anzhen Hospital. All these patients were admitted to the hospital because of coronary heart disease. We used duplex ultrasound to diagnose carotid stenosis according to guideline recommendations, with severe stenosis of the carotid artery (≥ 70% stenosis) defined as a combination of peak systolic velocity of 230 cm/s and an end-diastolic velocity of ≥ 100 cm/s or a peak systolic velocity ratio between the internal and common carotid artery of ≥ 4 [11]. If the patient has significant neurological symptoms or had a history of transient ischemic attack, stroke, or amaurosis fugax within 6 months, it is considered symptomatic carotid stenosis. Patients with carotid stenosis greater than 70% or with symptomatic lesions had a carotid enhanced computed tomography angiogram, and some of these patients underwent carotid angiography. We use coronary angiography to define the degree of coronary artery stenosis and select appropriate patients for bypass surgery according to guideline recommendations [12]. In our hospital, the indication for carotid revascularization combined with CABG is defined as a carotid artery diameter reduction of greater than 70% (asymptomatic) or symptomatic carotid stenosis. The exclusion criteria included patients who were aged less than 18 or more than 80 years old, patients with coagulation dysfunction, chronic carotid total occlusion, combined with vertebral artery or subclavian artery stenosis, history of stroke within 3 months, and general conditions that did not allow the patient to tolerate combined surgery, and patients who refused simultaneous surgery, patients with any other illness that impeded their ability to provide informed consent, and patients with previous open heart surgery. According to the above inclusion and exclusion criteria, 88 patients were finally included in this study (Fig. 1), 25 of the patients underwent combined CAS–CABG and 63 patients underwent combined CEA–CABG. All patients were admitted with preoperative antiplatelet therapy (Aspirin, 100 mg, Qd) for ≥ 2 days and postoperative Low Molecular Weight Heparin (100 U/kg, Q12h), with early resumption of Aspirin (100 mg, Qd) + Clopidogrel (75 mg, Qd) based on the patient's drainage and risk of bleeding. Other preoperative preparations were the same as for CABG surgery. All patients were treated with dual antiplatelet therapy for 1 year after surgery, Clopidogrel was discontinued after 1 year, and Aspirin was administered for life.

### Study design

In this single-center retrospective study, we analyzed the demographics, history of the disease, imaging findings, surgical procedures, and postoperative outcomes of 88 patients who underwent carotid revascularization combined with CABG. GE (USA) Vivid 7 and E9 ultrasound systems (M3S) were used for carotid ultrasound and echocardiography. For echocardiography, the patients were examined in the supine position. The parameters obtained included the degree of carotid stenosis, ejection fraction, and valve insufficiency. The type of surgery included CEA–CABG and CAS–CABG. All patients underwent carotid revascularization and CABG under the same anesthesia. All follow-up of the study came from clinical visits or telephone follow-up after discharge. The follow-up time for the early results was 30 days after discharge, and the deadline for the mid-term follow-up was March 1, 2022. All procedures involving human participants in this study complied with the Declaration of Helsinki (revised in 2013). The ethics committee of Beijing Anzhen Hospital approved the study (Institutional Review Board File 2014019). Since the study did not involve the specific personal information of patients, the ethics committee waived the need for the informed consent of each patient.

### Surgical techniques

After induction of general anesthesia, carotid artery revascularization was performed first. For the patients who underwent combined CEA–CABG, an oblique incision was made at the front edge of the sternocleidomastoid muscle on the affected side. After the blood vessels were separated, 50 mg heparin was applied to make the ACT reach more than 250 s. The internal carotid artery, external carotid artery, superior thyroid artery, and common carotid artery on the affected side were blocked. The common carotid artery was cut longitudinally, and the incision was extended to the distal end of the internal carotid artery and external carotid artery. After the common carotid internal carotid artery bypass was established with a shunt tube, the intima of the diseased blood vessel was removed, the wound was repaired with a patch after repeated flushing, and the wound was sutured after opening the artery in turn. In the patients who underwent combined CAS–CABG, all procedures were performed in the Hybrid Operating Room, the femoral artery was punctured with the Seldinger technique, an 8F arterial sheath was inserted, 3000 U heparin sodium was injected, a 5F pigtail catheter was used for ascending aortography, and a 5F VER catheter was placed in the innominate artery, common carotid artery and right subclavian artery to observe the vascular involvement. After 2000 U heparin sodium was injected into the artery, the cerebral protection device was released at the distal end of the internal carotid artery of the affected side, and the balloon was used to expand the lesion step by step. The stent was placed, the cerebral protection device was recovered, the catheter was removed, and the puncture port was closed with the suturing device. After carotid revascularization, the patient’s chest was opened in the middle of the sternum, and coronary artery bypass grafting was performed with or without cardiopulmonary bypass.

### Statistical analyses

We divided the selected patients into combined CAS–CABG and combined CEA–CABG groups according to the type of surgery. Continuous variables with a normal distribution are expressed as the mean ± standard deviation. Continuous variables without a normal distribution are expressed as the median (interquartile range). Categorical variables are expressed as numbers (percentages). The t-test, Wilcoxon test, and Pearson chi-square test were used to analyze the differences between the two groups for variables with a normal distribution, continuous variables without a normal distribution, and categorical variables. For the right-censored data, we used the Kaplan–Meier analytical method and log-rank test to compare mid-term survival and its 95% confidence interval and plotted the survival curve. We used univariate and multivariable Cox proportional hazards regression to evaluate independent risk factors affecting patient death and adjust for confounding factors. Variables with *P* < 0.10 in univariate analysis were included in multivariable Cox regression analysis. *P* < 0.05 was considered statistically significant [2-sided]. Statistical software R 4.1.0 was used for all analyses (http://www.R-project.org, the R Foundation).

## Result

### Perioperative characteristics

In our study, the average age of the patients was 65.32 ± 7.53 years. A total of 80.7% of the patients had asymptomatic carotid stenosis. Twenty-five patients received combined CAS–CABG, and 63 patients received combined CEA–CABG. The perioperative characteristics of the patients in the two groups are shown in Table 1. There were no significant differences between the two groups in the comparison of preoperative baseline data. In terms of the intraoperative details, there were no significant differences in the number of bridging vessels between the two groups (*p* = 0.09). In the combined CEA–CABG group, 6 patients underwent on-pump surgery and 3 patients underwent valve surgery at the same time. The total hospital stay of the combined CAS–CABG group was significantly shorter than that of the combined CEA–CABG group (*p* = 0.01).

**Table 1:** Perioperative characteristics of the two groups of patients

Variables	Combined CEA–CABG (n = 63)	Combined CAS–CABG (n = 25)	*P* value
Age (y), mean ± SD	65.1 ± 7.8	66.0 ± 6.9	0.617
Female sex, n (%)	14 (22.2)	1 (4.0)	0.083
BMI, medium (IQR)	25.3 (4.1)	25.4 (5.7)	0.817
NYHA class, n (%)			0.263
I	0	0	
II	36 (57.1)	11 (44.0)	
III	22 (34.9)	9 (36.0)	
IV	5 (7.9)	5 (20.0)	
Smoking, n (%)	31 (49.2)	13 (52.0)	0.813
Drinking, n (%)	12 (19)	5 (20.0)	0.919
Hypertension, n (%)	47 (74.6)	17 (68)	0.717
Diabetes mellitus, n (%)	25 (39.7)	13 (52.0)	0.416
Dyslipidemia, n (%)	7 (11.1)	5 (20.0)	0.452
Atrial fibrillation, n (%)	2 (3.2)	0	0.914
Previous stroke, n (%)	10 (15.9)	8 (32.0)	0.162
Previous MI, n (%)	17 (27.0)	8 (32.0)	0.835
Previous PCI, n (%)	4 (6.3)	2 (8.0)	0.782
Symptomatic carotid stenosis, n (%)	14 (22.2)	3 (12.0)	0.426
Carotid occlusion, n (%)	4 (6.3)	1 (4.0)	0.657
Unilateral carotid stenosis, n (%)	38 (60.3)	16 (64.0)	0.749
Angina pectoris, n (%)	24 (96.0)	60 (95.2)	0.867
Three-vessels disease, n (%)	57 (90.5)	23 (92.0)	0.823
Left main disease, n (%)	8 (12.7)	4 (16.0)	0.950
No. of bridging vessels, medium (IQR)	3 (1.0)	3 (0)	0.090
LVEF, medium (IQR)	62 (12.0)	63 (6.0)	0.696
On-pump, n (%)	6 (9.5)	0	0.177
Combined with valve surgery, n (%)	3 (4.8)	0	0.555
Intensive care unit stay (days), medium (IQR)	1 (1.0)	1 (0)	0.228
In-hospital stay (days), medium (IQR)	20 (9.0)	16 (9.0)	0.01*

*BMI* Body Mass Index, *NYHA* New York Heart Association, *LVEF* left ventricular ejection fraction, *MI* myocardial infarction, *PCI* percutaneous coronary intervention; values are presented as mean ± standard deviation or number (%) unless indicated otherwise; The degree of stenosis was measured using the North American Symptomatic Carotid Endarterectomy Trial (NASCET) method; *p < 0.05

### Early results

In terms of the early results (30 days after the operation), 3 patients (4.80%) died in the combined CEA–CABG group, 1 patient (1.60%) died of postoperative infarction, 1 patient (1.60%) died of malignant ventricular arrhythmia, and 1 patient (1.60%) died of respiratory and circulatory failure. No patient in the combined CAS–CABG group died or had a myocardial infarction, and one patient (4.00%) had an ischemic stroke 1 day after the procedure. CT of the head suggests multiple infarcts in the parietal and occipital lobes ipsilateral to the patient's carotid stenting procedure, and carotid ultrasound showed a clear signal for blood flow in the stent. No significant difference was found in the composite results between the two groups (odds ratio [OR], 1.20; 95% CI 0.12–12.12; *P* = 0.88). Table 2 summarizes the early results and incidence of adverse events of the two groups.

**Table 2:** Early or midterm outcomes of combined CEA–CABG and combined CAS–CABG

	Combined CEA–CABG (n = 63)	Combined CAS–CABG (n = 25)	OR (95% CI)	*P* value
Early term outcomes				
Death	3 (4.80)	0 (0)	–	–
Stroke	0 (0)	1 (4.00)	–	–
MI	1 (1.60)	0	–	–
Composite events	3 (4.80)	1 (4.00)	1.20 (0.12, 12.12)	0.88
Midterm outcomes				
Death	9 (14.30)	1 (4.00)	4.00 (0.48, 33.37)	0.20
Stroke	4 (6.30)	2 (8.00)	0.78 (0.13, 4.55)	0.78
MI	3 (4.80)	1 (4.00)	1.20 (0.12, 12.12)	0.88
Composite events	13 (20.60)	4 (16.00)	1.37 (0.40, 4.68)	0.62

*MI* Myocardial infarction, *CI* confidence interval, *OR* odds ratio

### Mid-term results

**Fig. 2:**
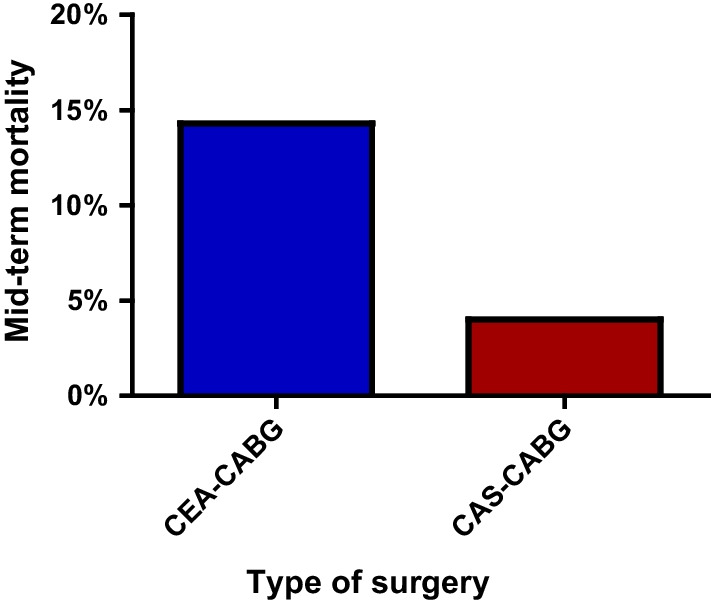
Mid-term mortality of the two groups; CABG, coronary artery bypass graft; CAS, coronary artery stenting; CEA, carotid endarterectomy

**Fig. 3:**
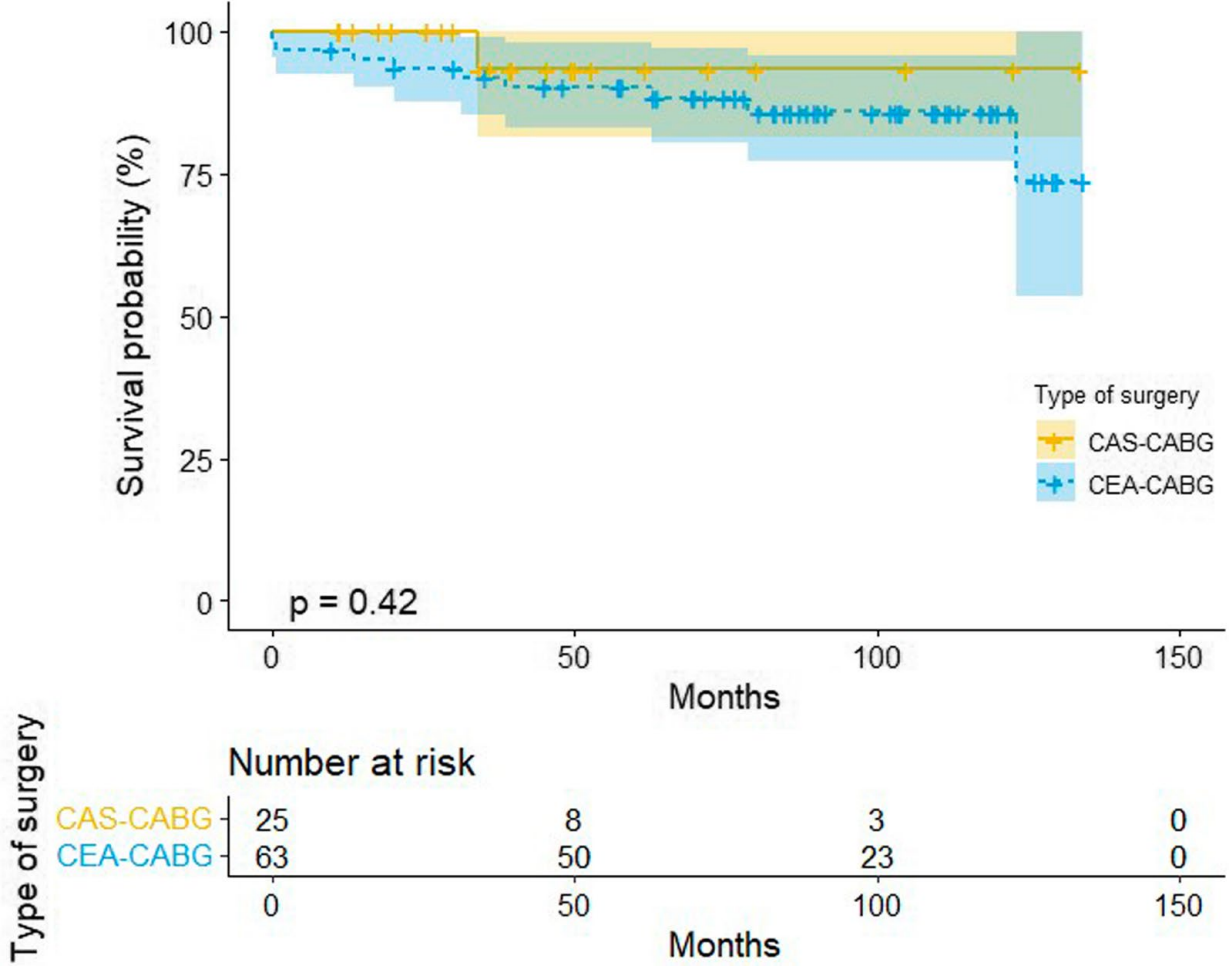
Kaplan–Meier curve: X line: follow-up time since surgery (months). Y line: rate of survival probability. CABG, Coronary artery bypass graft; CAS, coronary artery stenting; CEA, carotid endarterectomy

**Fig. 4:**
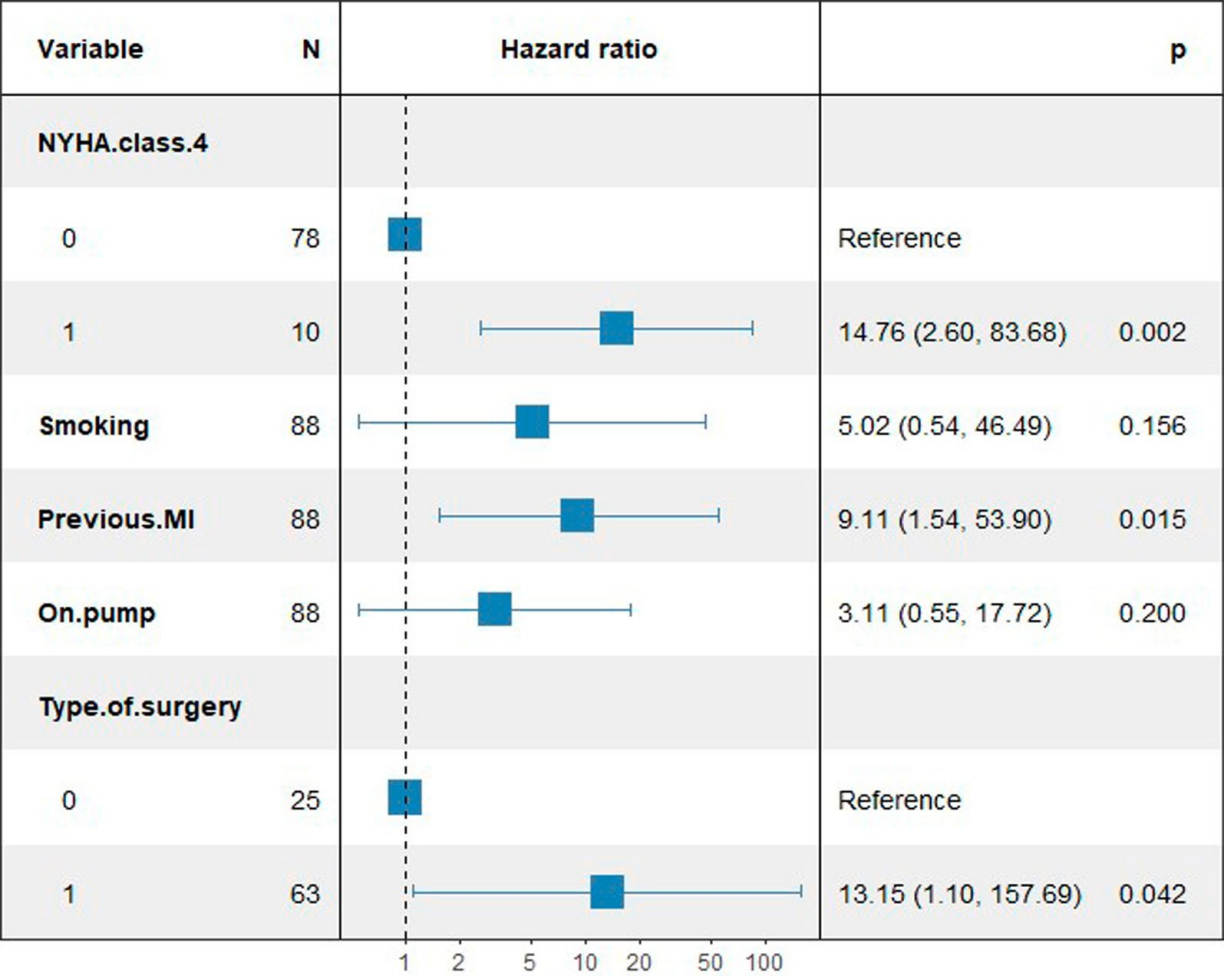
Adjusted multivariable Cox proportional hazards regression analysis for risk factors of mortality; NYHA, New York Heart Association; MI, myocardial infarction; Type of surgery 0 means CAS–CABG; Type of surgery 1 means CEA–CABG

During the median follow-up period of 6.69 years (IQR, 5.82–7.57 years), 9 patients (14.30%) died in the combined CEA–CABG group, while only 1 patient (4.00%) died in the combined CAS–CABG group (Fig. 2). Four patients (6.30%) in the combined CEA–CABG group had a stroke, of which two were ischemic strokes contralateral to carotid surgery, one was ischemic stroke ipsilateral to carotid surgery, one was a hemorrhagic stroke, and three patients (4.80%) had a myocardial infarction. Two patients (8.00%) in the combined CAS–CABG group had a stroke, of which one was an ischemic stroke ipsilateral to the carotid procedure, one was a hemorrhagic stroke, and one patient (4.00%) had a myocardial infarction. No significant difference was found in the median mortality (odds ratio [OR], 4.00; 95% CI 0.48–33.37; *P* = 0.20), stroke (odds ratio [OR], 0.78; 95% CI 0.13–4.55; *P* = 0.78), myocardial infarction (odds ratio [OR], 1.20; 95% CI 0.12–12.12; *P* = 0.88) or composite results (odds ratio [OR], 1.37; 95% CI 0.40–4.68; *P* = 0.62). The mid-term results are shown in Table 2. The Kaplan–Meier survival analysis showed that there was no significant difference in the mid-term total mortality between the two combined surgical groups (log-rank, *P* = 0.42; Fig. 3). We used the Cox proportional hazard regression model to evaluate the influencing factors related to death in the patients undergoing combined carotid revascularization and CABG. After the univariate analysis, New York Heart Association (NYHA) grade IV, smoking, previous myocardial infarction, and on-pump were included in the multivariate analysis. It was found that NYHA grade IV (hazard ratio [HR] 5.01, 95% CI 1.16–21.64, *p* = 0.03) and previous myocardial infarction (hazard ratio [HR] 5.43, 95% CI 1.01–29.29, *p* = 0.04) were independent risk factors for mortality in the patients undergoing combined surgery (Table 3). After incorporating the type of surgery into the multivariate analysis, we found that combined CEA–CABG surgery may be associated with a higher risk of death (HR, 13.15; 95% CI 1.10–157.69, *p* = 0.04; Fig. 4).

**Table 3:** Cox proportional hazards regression analysis for risk factors of mortality

Variables	Univariable		Multivariable	
	HR (95% CI)	*P* value	HR (95% CI)	*P* value
Age	1.01 (0.93, 1.10)	0.83	–	–
Female sex	0.40 (0.05, 3.32)	0.39	–	–
BMI	1.02 (0.85, 1.24)	0.82	–	–
NYHA class IV	5.19 (1.44, 18.75)	0.01*	5.01 (1.16, 21.64)	0.03*
Smoking	8.46 (1.06, 67.69)	0.04*	3.73 (0.40, 34.42)	0.25
Drinking	1.14 (0.24, 5.50)	0.87	–	–
Hypertension	1.55 (0.33, 7.35)	0.58	–	–
Diabetes mellitus	0.95 (0.27, 3.36)	0.95	–	–
Previous stroke	1.06 (0.22, 5.01)	0.94	–	–
Previous MI	10.04 (2.08, 48.57)	< 0.01*	5.43 (1.01, 29.29)	0.04*
Previous PCI	1.34 (0.17, 10.61)	0.78	–	–
Symptomatic carotid stenosis	1.31 (0.32, 5.37)	0.71	–	–
Unilateral carotid stenosis	1.52 (0.43, 5.31)	0.52	–	–
Left main disease	0.69 (0.09, 5.51)	0.73	–	–
No. of bridging vessels	0.75 (0.31, 1.86)	0.54	–	–
LVEF	0.95 (0.89, 1.02)	0.18	–	–
On-pump	4.18 (0.87, 20.14)	0.08	4.10 (0.71, 23.59)	0.11
Type of surgery	2.29 (0.29, 18.40)	0.44	–	–
Dual antiplatelet therapy after surgery	0.46 (0.13, 1.61)	0.23	–	–

*BMI* Body Mass Index, *NYHA* New York Heart Association, *LVEF* left ventricular ejection fraction, *MI* Myocardial infarction, *PCI* percutaneous coronary intervention, *HR* hazards ratio; *p < 0.05

## Discussion

Stroke is one of the most important complications during the perioperative period of CABG. The perioperative mortality of patients with stroke after CABG surgery is increased by 7.3 times [13]. Carotid artery stenosis has been proven to be an independent risk factor for perioperative stroke after CABG [14]. The AHA guidelines and expert consensus recommend carotid revascularization for symptomatic severe carotid stenosis or bilateral severe carotid stenosis to reduce the risk of stroke [15, 16]. The latest SVS guidelines recommend CEA surgery for asymptomatic patients with severe carotid stenosis at low surgical risk [11]. Recent studies have also shown that in patients with unilateral severe asymptomatic carotid artery stenosis undergoing CABG, carotid endarterectomy in stages or at the same time can reduce the risk of stroke [17]. Due to the lack of high-quality clinical research evidence, the treatment strategy of carotid revascularization combined with CABG is still controversial. Among the patients who received combined carotid revascularization and CABG in the United States, combined CEA–CABG was the most frequently used, followed by staged CEA–CABG and staged CAS–CABG. The study found that although patients undergoing CAS had more cardiovascular complications, the CAS–CABG strategy was still associated with the lowest in-hospital mortality [9]. A 10-year multicenter randomized controlled trial showed that there was no significant difference in the risk of perioperative stroke, myocardial infarction or death, and subsequent ipsilateral stroke between patients undergoing CAS and patients undergoing CEA. CAS can be used as an alternative to CEA [10]. The results of a meta-analysis showed that there was no significant difference in the prognosis between CAS–CABG on the same day and staged CAS–CABG, and the use of the simultaneous operation could allow the patients to avoid a second admission and the problem of anticoagulation between the two operations [18]. With the popularization of hybrid operation strategies, an increasing number of hospitals can perform combined CAS–CABG, but there have been no studies comparing the outcomes of combined CEA–CABG and combined CAS–CABG. This study found that there was no significant difference in survival, stroke, myocardial infarction, or combined adverse events between the two groups during the 30-day and mid-term follow-up, suggesting that combined CAS–CABG surgery can be a safe and effective treatment for patients with coronary heart disease complicated with severe carotid stenosis. NYHA grade IV and previous MI were independent risk factors for mid-term mortality. The results of the multivariate analysis suggest that CEA–CABG may be associated with a higher risk of death, but this still needs to be confirmed by a larger sample size study.

The average age of our patients was 65.32 ± 7.53 years old. The average age of the combined CEA–CABG group was 65.1 ± 7.8 years, and the average age of the combined CAS–CABG group was 66.0 ± 6.9 years. There was no significant difference between the two groups. In the United States, due to the limitation of medical insurance, CAS is usually performed in patients with a higher risk and, who are often older and have more cardiovascular complications [9]. However, in our center, we do not decide which carotid artery revascularization method to use according to the patient's age or complications. Therefore, in our study, there was no significant difference in the preoperative complications between the two groups. Although it is still controversial whether surgical intervention is recommended for asymptomatic carotid stenosis and the latest CABACS study and SPACE-2 study are currently limited by their sample size and do not yield high-quality results [19, 20]. Because our study was retrospective, we found it difficult to guarantee comparability between patients with severe carotid stenosis who underwent CABG without carotid revascularization and those who underwent the concurrent combined procedure, so we did not include patients without carotid revascularization for comparison in this study, but mainly compared the efficacy of different carotid revascularization modalities in the combined CABG procedure. Approximately 78.1–96% of the patients in the previous study had asymptomatic carotid stenosis [9, 21–23], and 80.7% of the patients in our study had asymptomatic carotid stenosis. The latest SVS guidelines recommend CEA for asymptomatic patients with severe carotid stenosis rather than drug therapy alone, especially since CABG itself increases the risk of stroke. In addition, we did not perform concurrent carotid revascularization in all asymptomatic patients with severe carotid stenosis, but only in those who were considered to be at high risk of stroke after comprehensive evaluation and who were willing to undergo concurrent surgery. It may be difficult to achieve complete uniformity of surgical intervention criteria in patients limited by retrospective studies, and we have only summarized the results of our part of the combined procedure, which is one of the limitations of this article. Only six of our combined procedures were performed with on-pump CABG, and the vast majority of CABG procedures at our center are off-pump CABG, especially in patients at high risk for stroke, and previous studies have shown that off-pump CABG significantly reduces the risk of stroke compared to on-pump CABG [24]. A total of 38.6% of our patients had bilateral carotid artery stenosis, but the stenosis on the other side was not severe. Therefore, all patients underwent unilateral surgery. Limited by the sample size and retrospective research methods, our study did not include the stenosis degree of the other side in this study, which is also one of the limitations of this study.

The main findings of this study are that both early and mid-term results of the combined CAS–CABG are similar to the combined CEA–CABG. After incorporating the type of surgery into the multivariate regression, the results of this study showed that combined CEA–CABG may be associated with a higher risk of death. We believe that this may be related to the progress of CAS surgery and the application of brain protection devices. Feldman et al. found that in the United States, even if the staged CAS–CABG strategy is performed in high-risk populations, this strategy is still associated with a lower risk of death compared with combined CEA–CABG [9]. In our study, we also found similar conclusions in combined surgery. In terms of the mid-term results, 9 patients (14.30%) in the combined CEA–CABG group died, while only 1 patient (4.00%) in the combined CAS–CABG group died. There was a significant difference in the absolute numbers between the two groups, although the difference was not statistically significant (*p* = 0.20). Multivariate Cox proportional hazards regression showed that combined CEA–CABG may be associated with a higher risk of death than combined CAS–CABG (HR, 13.15; 95% CI 1.10–157.69, *p* = 0.04). We believe that the results may be due to our limited sample size, resulting in an insufficient absolute number of endpoint events. Since there is no report on a study that evaluated combined CAS–CABG using a larger sample size at present, we speculate that with the gradual expansion of our study sample size in the future, there may be a significant difference in the mid-term death risk between the two combined surgery strategies. Bitao Xiang et al. found that combined CEA–CABG and staged CAS–CABG had a similar risk of death, stroke, and myocardial infarction in the mid-term outcome [21]. This study confirmed the efficacy of staged CAS–CABG. With the popularization of the hybrid surgery concept, an increasing number of centers can perform combined CAS–CABG. Our study confirmed the efficacy of the combined CAS–CABG strategy. Our study also found that NYHA grade IV and previous myocardial infarction were independent risk factors for death in patients undergoing simultaneous carotid revascularization combined with CABG, which suggests that we should be more careful in choosing a surgical strategy for such patients, especially whether choosing combined surgery.

Anticoagulation and antiplatelet therapy strategies are also important in patients undergoing carotid revascularization combined with CABG, and preoperative administration of antiplatelet therapy to patients may increase the risk of intraoperative and postoperative hemorrhage in CABG. Our center's experience has been to use aspirin preoperatively in all patients, either CAS–CABG or CEA–CABG, and in patients undergoing combined procedures our center's experience has been that preoperative discontinuation of aspirin is not necessary. Previous results from randomized controlled trials have confirmed that preoperative aspirin in patients undergoing CABG until the day of surgery does not increase the risk of bleeding [25]. The combined procedure also avoids the problem of anticoagulation and antiplatelet therapy in between the two procedures compared to staged procedures, where patients may face more complex thrombosis and bleeding problems. All patients were treated with dual antiplatelet therapy for 1 year after surgery, Clopidogrel was discontinued after 1 year, and Aspirin was administered for life. We used the above-mentioned antiplatelet therapy strategy for all patients after CABG alone and therefore did not increase the risk of postoperative bleeding due to the combined procedure. However, this strategy is only a summary of our clinical experience, and future anticoagulation and antiplatelet treatment strategies in combined surgery will require a larger sample of studies to provide high-quality evidence to guide clinical practice.

This study had some limitations. First, this was a retrospective study. There might be selection bias in the selection of patients, although our baseline data suggest that the two groups are comparable, there may be differences in patient selection between the two procedures, and different specialists may have different preferences for CEA and CAS, all of which may affect the final outcome. Second, the sample size of this study was small, especially for patients undergoing combined CAS–CABG, which limited the statistical efficiency. However, the number of combined CAS–CABG surgeries in other centers was also very limited [26], and we will continue to accumulate more cases. Finally, the study failed to carefully evaluate the degree of carotid artery stenosis and the impact of carotid artery stenosis on the other side in the patients in the two groups, but none of our patients had severe stenosis on the other side, thus not meeting the intervention indication. Our research is still ongoing. In the future, with the accumulation of more cases and the extension of follow-up, further research results will be reported.

## Conclusion

Combined CAS–CABG is a safe and effective treatment for patients with coronary heart disease complicated with severe carotid stenosis. The short-term and mid-term outcomes are similar to those of combined CEA–CABG and may be related to lower mid-term mortality, but this still needs to be confirmed by a larger sample size study. We also found that NYHA grade IV and previous myocardial infarction were independent risk factors for mid-term mortality.

## Data Availability

The datasets used and/or analyzed during the current study are available from the corresponding author upon reasonable request.

## Abbreviations

CABG: Coronary artery bypass graft
CAS: Carotid artery stenting
CEA: Carotid endarterectomy
BMI: Body Mass Index
NYHA: New York Heart Association
LVEF: Left ventricular ejection fraction
MI: Myocardial infarction
PCI: Percutaneous coronary intervention
CI: Confidence interval
OR: Odds ratio
HR: Hazards ratio
